# Simultaneous Single-Sample Determination of NMNAT Isozyme Activities in Mouse Tissues

**DOI:** 10.1371/journal.pone.0053271

**Published:** 2012-12-31

**Authors:** Giuseppe Orsomando, Lucia Cialabrini, Adolfo Amici, Francesca Mazzola, Silverio Ruggieri, Laura Conforti, Lucie Janeckova, Michael P. Coleman, Giulio Magni

**Affiliations:** 1 Department of Clinical Sciences (DISCO), Section of Biochemistry, Polytechnic University of Marche, Ancona, Italy; 2 Department of Agricultural, Food and Environmental Sciences (D3A), Polytechnic University of Marche, Ancona, Italy; 3 School of Biomedical Sciences, University of Nottingham, Medical School, Queen’s Medical Centre, Nottingham, United Kingdom; 4 The Babraham Institute, Babraham Research Campus, Babraham, Cambridge, United Kingdom; 5 School of Biology and Biotechnology, University of Camerino, Camerino (MC), Italy; Laurentian University, Canada

## Abstract

A novel assay procedure has been developed to allow simultaneous activity discrimination in crude tissue extracts of the three known mammalian nicotinamide mononucleotide adenylyltransferase (NMNAT, EC 2.7.7.1) isozymes. These enzymes catalyse the same key reaction for NAD biosynthesis in different cellular compartments. The present method has been optimized for NMNAT isozymes derived from *Mus musculus*, a species often used as a model for NAD-biosynthesis-related physiology and disorders, such as peripheral neuropathies. Suitable assay conditions were initially assessed by exploiting the metal-ion dependence of each isozyme recombinantly expressed in bacteria, and further tested after mixing them *in vitro*. The variable contributions of the three individual isozymes to total NAD synthesis in the complex mixture was calculated by measuring reaction rates under three selected assay conditions, generating three linear simultaneous equations that can be solved by a substitution matrix calculation. Final assay validation was achieved in a tissue extract by comparing the activity and expression levels of individual isozymes, considering their distinctive catalytic efficiencies. Furthermore, considering the key role played by NMNAT activity in preserving axon integrity and physiological function, this assay procedure was applied to both liver and brain extracts from wild-type and Wallerian degeneration slow (Wld^S^) mouse. Wld^S^ is a spontaneous mutation causing overexpression of NMNAT1 as a fusion protein, which protects injured axons through a gain-of-function. The results validate our method as a reliable determination of the contributions of the three isozymes to cellular NAD synthesis in different organelles and tissues, and in mutant animals such as Wld^S^.

## Introduction

**Figure 1:**
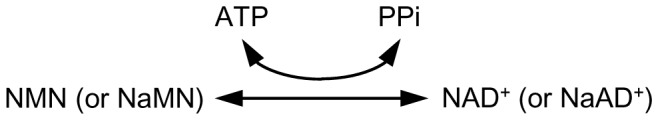
Overall NMNAT-catalyzed reaction. The mammalian NMNAT enzymes belonging to EC 2.7.7.1 are involved in both amidated and deamidated NAD biosynthesis pathways. NMN, nicotinamide mononucleotide; NaMN, nicotinic acid mononucleotide; NaAD^+^, nicotinic acid adenine dinucleotide or deamido-NAD.

The reaction catalysed by nicotinamide mononucleotide adenylyltransferase (NMNAT, EC 2.7.7.1) is universal in living cells and the sole known source of pyridine dinucleotides. Eukaryotic NMNATs transfer adenylate from ATP to nicotinamide mononucleotide or nicotinic acid mononucleotide, generating NAD or deamido-NAD, respectively, and PPi (Fig. 1). The reaction is easily reversible with an equilibrium constant close to unity [1] but the forward reaction is thought to be predominate *in vivo*, as NAD homeostasis fails without NMNAT [2], [3]. The reverse reaction is most likely limited by the physiological absence of PPi. In mammals, where the nicotinamide salvage pathway generates most NAD, the metabolic flux is mainly controlled by the preceding enzyme in the salvage pathway, nicotinamide phosphoribosyltransferase [4], which can be inhibited by FK866 [5], [6].

This highly conserved enzyme [7]–[10] has three isoforms in mammals arising from multiple genes and showing distinctive oligomerization properties, subcellular localization, and tissue distribution [11]–[14]. NMNAT1, NMNAT2 and NMNAT3 are predominantly localized in nuclei, Golgi membranes and mitochondria respectively. Human nuclear NMNAT1 is a homo-hexamer of 32 kDa subunits [15] and is the most widely-expressed isozyme and often the most abundant. The Golgi isozyme, NMNAT2, remains structurally undefined but was recently modelled *in silico* [16]. It is considered a 34 kDa monomer, most abundant in neuronal tissues [17]. NMNAT2 appears unstable and prone to oxidative denaturation due to its high cysteine content, two of which form an isoform-distinctive doublet which is palmitoylated to anchor NMNAT2 at the cytoplasmic surface of Golgi membranes [18]. Human mitochondrial NMNAT3 is a homo-tetramer of 28 kDa subunits [19], whose expression pattern show little overlap with NMNAT2, and which is less abundant than NMNAT1 [13], [14], [19]. One exception is human erythrocytes, which have a remarkably high level of NMNAT3 relative to NMNAT1 [20].

Each mammalian NMNAT isozyme has a characteristic divalent cation dependence, affinity, and specificity [9], [10]. They also show differential use of alternative substrates as ITP and GTP, or other purine nucleotide substrate analogs such as cancer chemotherapeutic tiazofurin monophosphate [13], [21]. Although the physiological relevance of these reactions remains unclear, the selective substrate specificity and metal ion dependence should allow the individual activities of the three human NMNATs to be determined in complex cell or tissue extracts [13]. This is an attractive tool to assess their relative contributions to NAD synthesis in different cell types, organelles, and responses to environmental stimuli, including any post-translational regulation.

The occurrence in mammalian cells of multiple, compartmentalized NMNAT isozymes strongly suggests that NAD biosynthesis is differentially regulated in different subcellular compartments [22], and the unequal distribution among mammalian tissues [13], [14] suggests tissue-specific functions, whose physiological and pathological implications require further study. Subcellular distribution is controlled by isoform-specific targeting and interaction domains (ISTIDs) [18], encoded by unique exons, which are absent in NMNATs from lower organisms and are dispensable for catalytic activity. Interestingly, the ISTIDs include post-translational modification sites, such as palmitoylation [23] or phosphorylation [24], likely to regulate compartmentalization of intracellular NAD pools. The roles of individual mammalian NMNATs, other NAD biosynthetic enzymes and their metabolic intermediates [22], are widely studied for example in DNA protection, regulation of gene expression, cell death, lifespan, and glucose metabolism [11], [12], [25], [26]. Although an isozyme discrimination method is available for the human enzymes [13], many of these studies use mouse tissue and the human assay cannot be directly transferred to other species where enzyme properties differ.

One important example is the role of NMNAT in axon survival and neuroprotection [12], [27]–[30]. The progressive breakdown of the distal segment of a severed axon, termed “Wallerian degeneration”, is a widespread, non-apoptotic death programme shared with some axonopathies [31]. A spontaneous mutation in mice, the Wallerian degeneration slow (Wld^S^) mutation [32], mutates NMNAT1, partly relocating it into axons. Understanding the neurodegenerative mechanism has identified molecular steps for pharmacological targeting [33], (Conforti et al., unpublished). Wld^S^ expression markedly delays the axonal degeneration after mechanical or chemical injuries, and also ameliorates several acute or chronic neuropathies morphologically converging into progressive axon degeneration [28], [34]. It has been proposed that Wld^S^ compensates for loss of the major endogenous axonal isozyme, NMNAT2, a labile protein that is essential for axon survival [29], [35], [36]. Thus, Wld^S^ represents an extra NMNAT isoform aberrantly present within the axoplasm of the mutant strain, allowing protection through a gain-of-function whose details are beginning to emerge (Conforti et al., unpublished). An important limitation for research on this and other significant topics relating to NMNAT has been the lack of methods to distinguish the effects of individual isozyme activities, especially in the absence of highly selective inhibitors. Most conclusions have been inferred from RNA and protein quantification experiments, but these are intrinsically unable to evaluate post-translational regulation and often limited by the low quality of currently available antibodies. Thus, a discrimination assay for mouse isozymes would fill an important gap.

To allow direct estimates of the three endogenous NMNAT isozyme activities in mouse tissues, as well as of the NAD-biosynthetic machinery where Wld^S^ acts, a novel assay has been developed and validated for simultaneous activity determination of all known NMNAT isozymes. Its application to both brain and liver extracts from Wld^S^ mice illustrates the potential for studying isoform-specific biochemistry in mouse tissues, thus facilitating future studies of how NMNAT isozymes respond individually to disease, injury or ageing.

## Methods

### Ethics Statement

Procedures using live animals were authorised under Project Licence 80/2254 that was approved both by the Babraham Research Campus Animal Welfare, Experimentation and Ethics Committee (AWEEC) and the UK Home Office. Animals were humanely killed using approved (Schedule 1) methods.

### Materials

Ni-NTA Superflow and TALON® metal affinity resins were purchased from Qiagen and Clonetech, respectively. PD-10 Sephadex™ columns were from GE Healthcare. *E. coli* strains TOP10F’ and BL21(D3), SuperScript™ II Reverse Transcriptase, and Platinum® SYBR® Green qPCR SuperMix UDG were from Invitrogen. Protein Assay kit and Taq-polymerase iProof^TH^ High-Fidelity were from Bio-Rad. The pET28 expression vectors were from Novagen. Other chemicals were from Sigma.

### Mouse Genotypes and Tissue Extract Preparation

Livers and brains from wild-type (C57BL/6) and mutant Wld^S^ mouse (C57BL/Wld^S^) were obtained from a breeding colony purchased from Harlan Laboratories (UK). Collected tissues were immediately snap-frozen in liquid nitrogen and stored at −80°C until the time of processing. Frozen mouse tissues were dipped into liquid nitrogen and ground by mortar and pestle to a fine powder. Thereafter, weighed tissue aliquots were extracted with perchloric acid for HPLC determination of endogenous NAD levels [37], using cAMP as an internal standard for recovery calculation. Alternatively, for activity assays, they were resuspended in 10 vol of 50 mM HEPES/KOH buffer, pH 7.5, 20 mM NaF, freshly supplemented with 1 mM dithiothreitol (DTT), 1 mM phenylmethylsulfonyl fluoride (PMSF), and 0.02 mg/mL leupeptine, antipain, chymostatin, pepstatin, and aprotinin. After gentle thawing on ice, each homogenate was sonicated 3 times at 50 watts (30 sec each with 0.5-sec impulse) with 1-min intervals on ice, and treated with Chelex-100 resin to remove interfering endogenous metal ions. In this step, pre-swollen Chelex-100 resin, washed twice with ice-cold distilled water just before the use, was gently mixed at 1∶ 3 vol ratio and quickly removed by mild centrifugation. Each metal-free supernatant was assayed to determine protein concentration (Bio-Rad Protein Assay kit) and immediately used for the discrimination assay (see below).

### Cloning and Bacterial Overexpression

Full-length open reading frames encoding *Mus musculus* NMNAT (mNMNAT) isoforms 1 (855 bp, GenBank AY679721), 2 (921 bp, GenBanK BC089007), 3 (756 bp, GenBanK BC005737), and Wld^S^ (1119 bp, GenBanK AF260924), were amplified by standard high-fidelity PCR from commercial plasmids. The primers used, carrying restriction overhangs for directional cloning into the polylinker region of pET28 vectors, are listed in supplemental Table S1. Directional cloning was performed at *Nde*I/*Eco*RI of pET28c for mNMNAT1, at *Nde*I/*Hind*III of pET28c for both mNMNAT2 and mNMNAT3, and at *Nhe*I/*Xho*I of pET28b for Wld^S^. Owing to this cloning strategy, the recombinant proteins were all fused to N-terminal His-Tag tails, *i.e.* MGSSHHHHHHSSGLVPRGSH for the three mNMNATs, and MGSSHHHHHHSSGLVPRGSHMAS for Wld^S^. The resulting plasmid constructs were replicated into *E. coli* TOP10F’, verified by sequencing for their exact match with database deposited sequences, and individually transformed into *E. coli* BL21(D3) for protein expression. After transformation, single colonies from kanamycin-selective plates were inoculated in 10 mL Luria-Bertani medium supplemented with 50 mg/L kanamycin, and grown at 37°C overnight under rotary shacking (200 rpm). About 5 mL of each pre-culture was inoculated in 250 mL fresh medium without antibiotic, and grown as before but at the temperature of 28°C to prevent or minimize inclusion bodies formation. At middle exponential phase (OD600 ∼0.8, usually 3–4 h incubation), 1 mM isopropylthio-β-galactoside was added to each culture and the induction was prolonged for additional 3 h at 28°C. Cells were finally collected by mild centrifugation, washed twice with PBS, and stored at −80°C.

### Purification of Recombinant His-tagged Proteins

All purification steps were performed at 4°C. Harvested bacterial cells expressing either mNMNAT1, or mNMNAT2, or mNMNAT3, or Wld^S^ recombinant species, were lysed by French Press at 18,000 psi after resuspension in 10–15 mL lysis buffer composed by 50 mM Na-phosphate, pH 7.0, 300 mM NaCl, 5 mM 3-(3-cholamidopropyl)dimethylammonium-2-hydroxy-1-propanesulfonate (CHAPSO) (for mNMNAT1 and mNMNAT2), or 50 mM HEPES/KOH, pH 7.5, 500 mM NaCl, 5 mM CHAPSO (for mNMNAT3 and Wld^S^), in either case freshly supplemented with 1 mM PMSF, 1 mM tris(2-carboxyethyl)phosphine (TCEP), and 0.05 mL/g cell pellet of protease inhibitor cocktail (Sigma #P8465). After brief incubation in the presence of lysozyme (1 mg/mL) and DNAse (10 µg/mL), the protein suspensions were clarified by centrifugation at 20,000×*g* for 30 min. Thereafter, the His-tagged mNMNAT3 and Wld^S^ species were purified by Ni-NTA affinity chromatography, carried out onto pre-packed columns (0.5–1 mL resin) equilibrated with 50 mM HEPES/KOH, pH 7.5, 500 mM NaCl, 1 mM TCEP, 1 mM PMSF. The washing and elution steps were carried out using 20 mM and 200 mM imidazole, respectively. Instead, the His-tagged mNMNAT1 and mNMNAT2 species were purified by TALON affinity chromatography, performed in 50 mM Na-phosphate buffer, pH 7.0, 300 mM NaCl, 1 mM TCEP, 1 mM PMSF. In this case, the washing and elution steps were carried out using 20 mM and 150 mM imidazole, respectively. Collected fractions from each chromatography were analysed by SDS-PAGE and assayed for their total NMNAT activity (see below). Active and homogeneous fractions were pooled and quickly desalted on PD-10 columns against 50 mM HEPES/KOH buffer, pH 7.5, 20% glycerol, 1 mM TCEP. Enzyme stability in solution was checked after storage in aliquots at either 4°C or −20°C. Before their use in discrimination assays, thawed samples were treated with Chelex-100 resin as above described (see *Tissue extract preparation*).

### NMNAT Activity Assays

Activity rates were measured by a C18-HPLC method [13], [37], based on measurement of the product formed by the NMNAT-catalyzed reaction, *i.e.* NAD or its analogs (respectively, nicotinamide hypoxanthine dinucleotide from ITP and nicotinamide guanine dinucleotide from GTP). Rates were calculated as tangent lines in the linear region of plots of product accumulation versus time. Kinetic parameters were measured as described [13] under 15% maximum consumption of both substrates concentration. One unit (U) of NMNAT activity refers to the enzyme amount catalysing 1 µmol/min product formation at 37°C.

The assays discriminating for individual isozyme activity (discrimination assays) are based on isozyme-selective metal ion dependence. The reference assay mixture (0.4 mL final volume) contained 30 mM HEPES/KOH, pH 7.5, 0.6 mg/mL BSA, 25 mM MgCl_2_, 20 mM NaF, 1 mM DDT, 1 mM both NMN and ATP, and either ∼1 mg/mL protein tissue extract or 0.15–2.5 µg/mL each pure recombinant isozyme. The above assay, referred as *“A”*, was used to measure total NMNAT activity, *i.e.* the “reference” activity value for all different mNMNAT isoforms. For isozyme discrimination, the additional mixtures *“B”*, “*C”*, and “*D”* were set replacing 25 mM MgCl_2_ with either 50 µM MgCl_2_ (*B*) or 1.5 mM ZnCl_2_ (*C*) or 4 mM CoCl_2_ (*D*). The assays under condition “*D*” were carried out in the absence of DTT. Suitable parameters for subsequent calculation were obtained by parallel assaying of each recombinant isozyme under the four conditions above. Then, reaction rates obtained from “*B*”, “*C*”, and “*D*” were divided each by the reference activity value obtained from “*A*”. The resulting reaction rate ratios, *i.e.* the coefficients b1, b2, b3; c1, c2, c3; d1, d2, d3, were substituted in the following system of three linear equations:

Tissue NMNAT activity (condition assay “*B*”) = (b1·X)+(b2·Y)+(b3·Z).

Tissue NMNAT activity (condition assay “*C*”) = (c1·X)+(c2·Y)+(c3·Z).

Tissue NMNAT activity (condition assay “*D*”) = (d1·X)+(d2·Y)+(d3·Z).

where X, Y, and Z represent the true enzymatic activities of mNMNAT1, mNMNAT2, and mNMNAT3, respectively, in the complex mixture. Solution of the matrix based on the Cramer’s rule [38] yields the actual activity values X, Y, and Z as if they were measured under reference condition “A”. Matrix calculation was carried out by Microsoft Excel and, specifically, by using the functions MDETERM(array), MINVERSE(array) and MMULT(array-1,array2), were “array” is the matrix of coefficients b1, b2, b3; c1, c2, c3; d1, d2, d3; “array-1“ is the transposed matrix of “array” obtained by the function MINVERSE(array), and “array2” is the vector obtained from tissue NMNAT activity values under conditions “*B*”, “*C*”, and “*D*”. The whole procedure including a numerical example of the matrix substitution calculation is detailed in Supporting Information files: Methods S1, Table S2, and Methods S2.

### Evaluation of mRNA Expression

Total RNA was extracted from frozen tissue samples by using TriSure (Bioline) according to the manufacturer’s instructions. First strand cDNA synthesis was performed using SuperScript™ II Reverse Transcriptase in the presence of 1 µg total RNA and oligo(d)T primer. Quantitative real time PCR was performed using Platinum® SYBR® Green qPCR SuperMix UDG and the primer pairs described in supplemental Table S1. The PCR efficiency for each set of primers was previously established by analysing serial dilutions of cDNA. All measurements were made at least in duplicate and the relative expression levels of the three mNMNATs were each normalized to the housekeeping gene β-actin [39].

## Results

### Comparative Kinetic, Catalytic Studies, and Selection of Suitable Discrimination Assay Conditions

**Figure 2:**
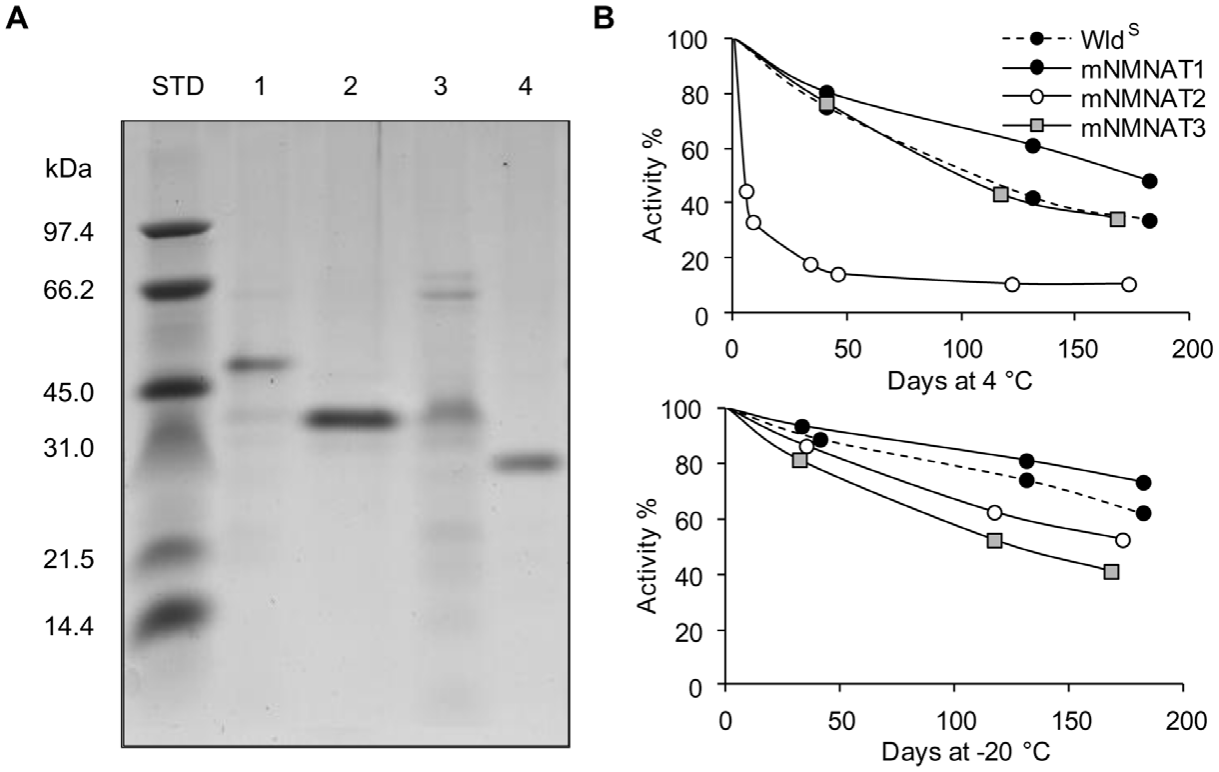
Bacterial overexpression and stability of recombinant isozyme preparations. Panel A) SDS-PAGE analysis of affinity purified His-tagged recombinant proteins. Gel lanes were loaded with standard protein markers of indicated mass values and 3–4 µg each of Wld^S^ (1), mNMNAT1 (2), mNMNAT2 (3), or mNMNAT3 (4) final enzyme preparation. ProtParam predicted masses of recombinant species are 44001 (1), 34518 (2), 36668 (3), and 29866 (4) daltons. Panel B) Stability of each preparation at 4°C and −20°C in a buffer solution composed by 50 mM HEPES/KOH, pH 7.5, 20% glycerol, and 1 mM TCEP.

Similar to the previously reported human method [13], our study of mNMNATs began using recombinant His-tagged enzymes obtained after bacterial overproduction. Indeed, sequence identity between mouse and human NMNAT counterparts is high, *i.e.* ∼80% for isoforms 1 and 3, and as high as 99% for isoform 2, with only four substitutions out of 307 total residues (not shown). After bacterial expression and one-step affinity chromatography, the different purifications yielded similar results and recovery values. Typically starting from 250 mL induced bacterial cultures, 50–80 mg protein and 0.1–0.4 U/mg total NMNAT activity were obtained in the soluble crude bacterial extract, while 0.5–2 mg protein with ∼80% activity yield were recovered in the purified enzyme preparations. Under the reference assay condition “A” (see Methods), the average specific activity value for both mNMNAT1 and Wld^S^ final preparations was ∼30 U/mg, while ∼5 U/mg and ∼2.5 U/mg were observed, respectively, for mNMNAT2 and mNMNAT3. Each final preparation also showed a protein band of the expected molecular mass after SDS-PAGE (Fig. 2A). Their stability in solution was later assessed after storage at 4°C and −20°C in an appropriate buffer at pH 7.5 containing both glycerol and TCEP as a reducing agent. As shown in Figure 2B, only mNMNAT2 at 4°C was rapidly inactivated, while similar stability at both temperatures was observed in all other cases, with activity losses ranging from 27% to 66% after six month storage. The comparable stability at –20°C of all our preparations (Fig. 2B, bottom) indicated this condition is optimal for long term storage of these expressed isozymes.

Further kinetic and catalytic characterization was conducted to identify feasible conditions to discriminate the activity of individual mouse isozymes in complex mixtures, in comparison with those reported for human NMNATs [13]. Inclusion of the Wld^S^ chimera was of potential relevance for the study of its individual contribution to neuroprotection.

Similar to human NMNATs [13], the N-terminal His-Tag did not affect the activity of the recombinant mouse enzymes. Their catalytic parameters (*K*_m_, *k*_cat_, and catalytic efficiency *k*_cat_/*K*_m_) for both NMN and ATP substrates were calculated from nonlinear fitting on primary plots. As summarized in Table 1, mNMNAT1, mNMNAT2, and mNMNAT3 showed substrate affinities in the micromolar range and *k*_cat_ values comparable to the corresponding human isozymes. The catalytic efficiencies were also similar, with the order mNMNAT1> mNMNAT2> mNMNAT3. The kinetic behaviour of the Wld^S^ chimera was remarkably superimposable to that of mNMNAT1, indicating that the N-terminal sequence of Wld^S^ mutation (Ube4b fragment [40], [41]) does not interfere with the enzyme catalysis.

**Table 1:** Kinetic parameters of mouse and human recombinant NMNAT isozymes.

	ATP			NMN		
	*K*_m_ (µM)	*k*_cat_ (s^−1^)	*k*_cat_/*K*_m_ (s^−1^M^−1^)	*K*_m_ (µM)	*k*_cat_ (s^−1^)	*k*_cat_/*K*_m_ (s^−1^M^−1^)
Wld^S^ (^a^)	29.6±1.8	25.5±0.4	8.6×10^5^	32.2±1.2	25.8±0.4	8.0×10^5^
mNMNAT1 (^a^)	33.5±5.1	15.0±0.7	4.5×10^5^	25.2±5.6	15.5±1.3	6.1×10^5^
mNMNAT2 (^a^)	82.0±15.1	2.33±0.2	0.3×10^5^	38.5±13.5	2.87±0.5	0.7×10^5^
mNMNAT3 (^a^)	39.0±5.9	0.74±0.04	0.2×10^5^	117.6±4.6	0.75±0.02	0.1×10^5^
human NMNAT1 (^b^)	58.5±4.2	53.9±1.4	9.2×10^5^	22.3±2.6	53.8±3.4	24.1×10^5^
human NMNAT2 (^b^)	88.9±18.3	8.8±0.4	1.0×10^5^	21.3±2.7	8.8±0.3	4.1×10^5^
human NMNAT3 (^b^)	42.1±5.8	2.5±0.3	0.6×10^5^	66.2±8.5	2.5±0.2	0.4×10^5^

(^a^) Data calculated in this work by using substrate concentrations in the range 25–175 µM for ATP and 5–100 µM for NMN; (^b^) data from ref [13].

**Figure 3:**
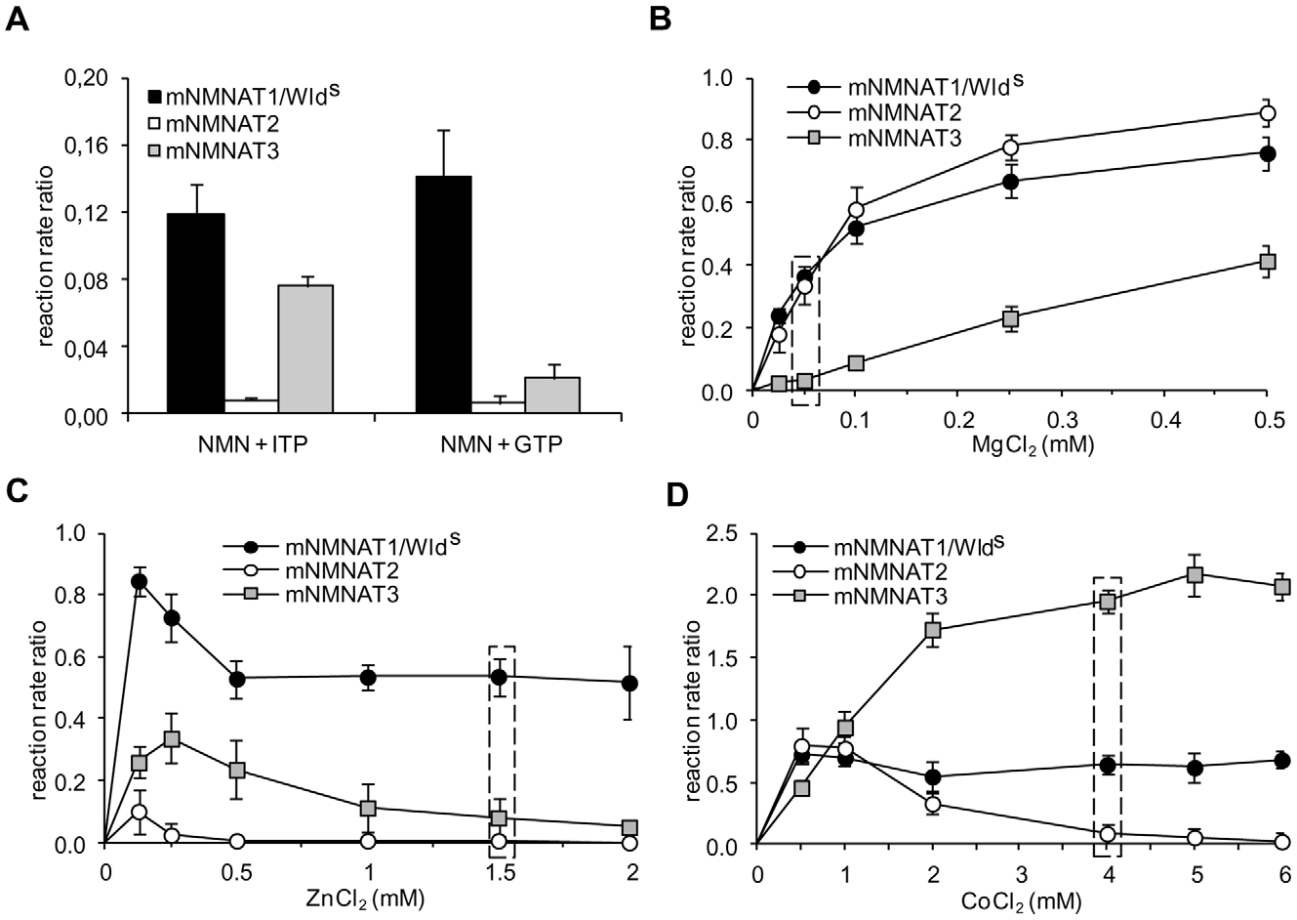
Use of alternative 5′-triphosphate purine substrates and metal ion dependence of recombinant isozymes. All values are averages (+/− SD) from triplicate HPLC analyses and referred as reaction rate ratios relative to the reference activity (condition “A”, see Methods). Panel A) Individual isozymes were assayed in the presence of 1 mM ITP or GTP substrates, either replacing 1 mM ATP in the standard assay. Panels B, C, and D) Individual isozymes were assayed at several concentrations of chloride salts of indicated metal ions, each replacing 25 mM MgCl_2_ in the standard assay. The assays with CoCl_2_ were also performed in the absence of DTT. mNMNAT1 and Wld^S^ data are combined because these were indistinguishable in all conditions tested. Boxed data points refer to the metal ion concentrations selected for discrimination in the present work, *i.e.*, 50 µM MgCl_2_ (B), 1.5 mM ZnCl_2_ (C), and 4 mM CoCl_2_ (D).

The activity discrimination of human NMNATs was based on parallel determination under assay conditions ensuring only one isozyme form is active at a time, *e.g.* by using high millimolar Zn^2+^ to select for isoform 1, low millimolar Mg^2+^ for isoform 2, and ITP for isoform 3 [13]. The behaviour of mouse recombinant isozymes under these conditions is summarized in Figure 3. In marked contrast to human NMNAT1, mNMNAT1 and Wld^S^ were able to use both ITP and GTP, supporting reaction rate ratios of 0.12 and 0.14, respectively, relative to the reference activity in the presence of ATP (Fig. 3A). Conversely, closely resembling their human counterparts, mNMNAT2 activity was barely detectable using both alternative substrates, while mNMNAT3 showed a clear preference for ITP with respect to GTP. Furthermore, in the presence of various concentrations of the chloride salts of both Mg^2+^ and Zn^2+^ (Fig. 3B and C), the resulting metal-dependence of mNMNATs was largely similar to the corresponding human isozymes [13], with the exception of mNMNAT1 and Wld^S^, whose Mg^2+^-dependence was superimposable with mNMNAT2 (Fig. 3B). These results show that the structurally-related mNMNAT1 and Wld^S^ enzymes are catalytically indistinguishable in all conditions tested. In addition, they clearly evidence remarkable catalytic differences between mice and humans regarding the sole isoform 1 that significantly appears more flexible in mice in the acceptance of ITP and Mg^2+^. These unexpected findings prevent utilization of our previous human protocol for mouse and prompted us to develop an alternative.

In subsequent experiments, we repeatedly observed more than one mNMNAT active at a time under a wide range of assay conditions tested, including variation of pH, ionic strength, temperature, and use of alternative substrates or metal cofactors other than those reported in Figure 3 (not shown). This difficulty in identifying conditions where only one isozyme was active, prompted us instead to develop a method to calculate the contribution of each isozyme using measurements made under three different conditions. Using different metal ion species and concentrations, we chose conditions that select for or against the activities of each isoform, even though other isoforms remained partially active (Fig. 3). This allowed us to generate three simultaneous equations whose unknowns are the activities of the three isozymes (see below). Two of the selected conditions, 50 µM Mg^2+^ (Fig. 3B, condition “*B*”) and 1.5 mM Zn^2+^ (Fig. 3C, condition “*C*”), are similar to those adopted in the previous human discrimination assay [13]. The third condition, 4 mM Co^2+^ (Fig. 3D, condition “*D*”) was selected because it supports a very high activity for isozyme 3 and, to a lesser extent, for isozyme 1. Furthermore, when Co^2+^ was used as cofactor, DTT was omitted because it interfered with the activity of all three mNMNATs leading to reaction rate reduction (not shown). In these assays, metal-free enzyme preparations obtained by Chelex treatment were used (see Methods), all displaying a maximum of 5% residual activity in the absence of metals (not shown).

### Calculation of Isozyme Activities in Mixtures

As the number of independent assay conditions is equal to the number of isozymes, the three simultaneous equations can be solved by Cramer’s rule [38]. To our knowledge, this approach has not previously been used to determine individual activities of multiple isozymes in unknown mixtures. In brief, NMNAT activity under condition “A” (25 mM MgCl_2_) provides a fixed reference point, and the reaction rate ratio relative to this value is obtained for each mouse isozyme using pure recombinant proteins under conditions “B”, “C”, and “D”. This indicates the nine coefficients reflecting the activity ratio exerted at each condition by each isozyme (see Methods). In parallel, the tissue NMNAT activity values under the same three discrimination conditions are measured. These latter values equal the sum of three distinct contributions, one by each isozyme, referred as the isozyme activity sought, multiplied by its coefficient (the reaction rate ratio). In this way, once all nine coefficients and the three tissue NMNAT activities are known, the three linear equations described in Methods can be generated, and a substitution matrix calculation based on Cramer’s rule can be easily applied to determine the three isozyme activities, *e.g. via* a pre-formatted MS Excel spreadsheet (see Methods S2). In Excel, the coefficients are arranged as a matrix to calculate its own inverse and the corresponding determinant; the tissue NMNAT activity values are then arranged as a vector to be multiplied by the inverse matrix above, thus yielding the three unknowns (X, Y, and Z). The system has a unique solution only if the matrix determinant is not zero. The three resulting values X, Y, and Z, represent the individual isozyme contributions to total NAD formation in the original native mixture under the “reference” condition “A”, that is used to assay total NMNAT activity in extracts from normal and pathological tissues.

### Analytical Validation

Validation of the proposed method was first carried out *in vitro* as previously done for human isozymes [13], using mixtures reconstituted from the three pure recombinant isozymes. Firstly, similar activities of each isozyme were mixed, as measured under condition “A” (reference condition, Table 2) and these known values compared to individual values from the matrix calculation (X, Y, and Z). The same reagent stocks were used for all assays to minimize experimental variability. The fit ranged from −13% to +7% suggesting this method provides reasonably accurate estimates of the contribution of each isozyme (Table 2).

**Table 2:** In vitro discrimination of mNMNAT activities (mU).

	Reference condition	Discrimination conditions				
	A	B	C	D	Matrix-calculated	Error %
mNMNAT1	1.28	0.72	0.50	0.84	1.12	−12%
mNMNAT2	1.85	0.02	0.63	0.14	1.98	+7%
mNMNAT3	1.64	0.19	0.05	3.21	1.42	−13%
MIXED	(4.77)^b^	0.81	1.15	3.62	(4.51)^b^	−5%

Table shows data from a typical discrimination experiment where the three recombinant mouse isozymes have been assayed both separately and after mixing in a reconstituted sample. The four assay conditions A, B, C, and D are described both in the text and in Methods. On the right, respectively, the activity values resulting after matrix calculation and the percent errors with respect to the measured values under reference condition “A”. (^b^) values in brackets are the sum of individual isozyme activities.

We then simulated biological variability by mixing unequal isozyme amounts ranging from a minimum of 0.54 mU up to a maximum of 2.40 mU as measured under reference condition “A” (Table 3). The results of four such independent experiments are shown in Table 3: the various isozyme amounts mixed yielded individual matrix-calculated values with percent errors ranging from −8% to +15%. This indicated sufficient statistical accuracy, regardless to the different proportions of isozymes mixed.

**Table 3:** In vitro discrimination of mNMNAT activities (mU).

	Experiment I		Experiment II		Experiment III		Experiment IV	
	Ref. cond. A	Error %	Ref. cond. A	Error %	Ref. cond. A	Error %	Ref. cond. A	Error %
mNMNAT1	1.33	−5.1%	0.67	−1.4%	1.60	+2.5%	1.43	+15%
mNMNAT2	0.54	+4.7%	1.27	−8.1%	1.37	−6.9%	2.40	−5.1%
mNMNAT3	1.68	−3.1%	1.99	+13%	2.04	−3.0%	0.65	+2.8%

Summary of results from four discrimination experiments, each carried out independently according to the scheme in Table 2.

**Figure 4:**
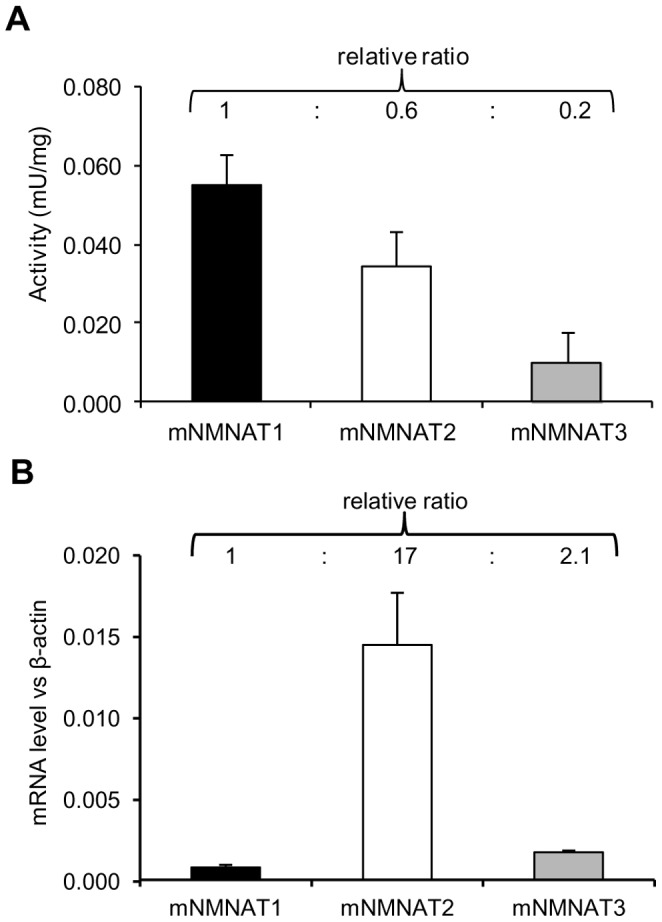
Comparative activity and expression profiles of individual mNMNATs in wild-type mouse brain. Tissues were collected from 24 month old animals. Panel A) Activity of mNMNAT isozymes from discrimination assay. Panel B) Real-time quantitative RT-PCR analysis. Relative mRNA expression levels are normalized against β-actin. Data are averages (+/− SD) of triplicate experiments.

Further validation of the proposed assay was done directly on a mouse tissue extract and compared to mRNA expression following parallel protein and RNA extraction (Fig. 4). Wild-type mouse brains were sagittally divided in the two cerebral hemispheres, to be used for either activity discrimination assays or quantitative RT-real time PCR. Specific activities of the three isozymes were obtained as described by assaying tissue extracts and pure recombinant mNMNATs in parallel, and relative expression levels normalized to β-actin. In these experiments, either right or left hemispheres were taken randomly, to be used for parallel protein and RNA extraction.

The sum of the three individual isozyme activities obtained from matrix calculation was in good agreement with the experimental value of 0.092±0.009 mU/mg for total NMNAT activity measured under reference condition “*A*” (25 mM MgCl_2_). Interestingly, total activity was almost entirely accounted for by mNMNAT1 and mNMNAT2 alone (Fig. 4A). The differences between activity and expression profiles are consistent with the differential kinetic properties and protein stabilities of the individual isozymes. For example, mNMNAT1 has a turnover number ∼6 and ∼20 times higher than mNMNAT2 and mNMNAT3 respectively (see *k*_cat_ values in Table 1), and mNMNAT2 is known to be a much less stable protein than either mNMNAT1 or mNMNAT3 [36] so its steady state protein level is likely to be lower than suggested by mRNA. Post-translational modification is another potential source of difference between activity and mRNA profiles.

### Discrimination Assay on Wild-type and Wld^S^ Mutant Mouse Tissues

**Figure 5:**
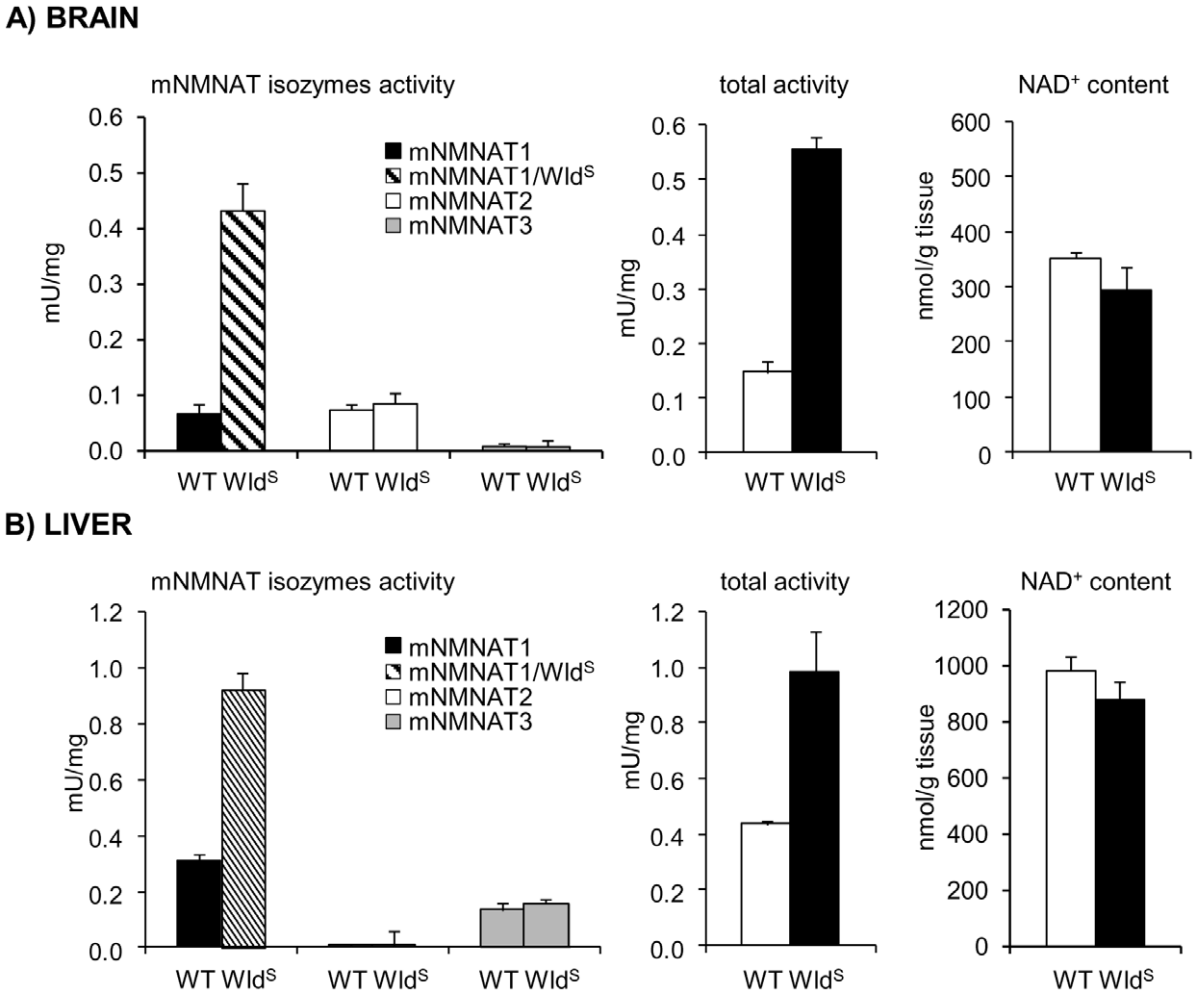
Activity profiling of mNMNAT isozymes in brain and liver from wild-type mouse and Wld^S^ mutant. Tissues were collected from 1 month old animals. The isozymes activities, measured by the described discrimination assay procedure, are color-coded as indicated (see graph legend). Tissue contents of total NMNAT activity and NAD, respectively determined under reference assay condition “A” and by HPLC (see Methods), are also shown. Each bar represents the average (+/− SD) from at least a triplicate experiment.

Application of the discrimination assay to wild-type brain and liver tissues revealed clearly distinct isozyme activity patterns in the corresponding extracts. mNMNAT1 (45%) and mNMNAT2 (50%) provided most activity in brain, while in liver mNMNAT1 (67%) and mNMNAT3 (30%) were most active (Fig. 5). In each case, the third isozyme activity, mNMNAT3 in brain and mNMNAT2 in liver, was barely detectable. Thus, these two tissues show widely differing and almost mutually exclusive patterns of NMNAT2 and NMNAT3 activity in keeping with overall predictions from reported expression data [12]–[14].

Parallel activity discrimination analyses were also carried out on brain and liver extracts from C57BL/Wld^S^ mutant mice, which constitutively express the axon-protective protein chimera Wld^S^, endowed with NMNAT1 activity [40], [41]. As a result, only the activity accounted by isoform 1, a combination of mNMNAT1 and Wld^S^ contributions, was found to be 3–6 times higher than in the corresponding wild type tissues (Fig. 5), while no statistical difference for the two other isozymes was observed (Student's t-test *p* values ≥0.1). Furthermore, as expected, no statistical alteration on NAD tissue content was observed in Wld^S^ mutant with respect to wild-type (Student's t-test *p* values ≥0.1).

## Discussion

In this work, a novel method for simultaneous activity discrimination of NMNAT isozymes in complex mixtures, like mouse tissue extracts, has been developed to study the compartmentalized cellular NAD biosynthesis and its role in physiological and pathological processes. The preliminary kinetic and catalytic characterization of mNMNATs, carried out *in vitro* by using recombinant isozymes, revealed properties broadly similar to their human counterparts, consistent with the structural similarities, as also recently reported by others for mNMNAT2 [42]. A known exception in mouse is NMNAT1 which accepts the alternative substrate ITP as well as low micromolar Mg^2+^ ions. Wld^S^ and mNMNAT1 were catalytically and kinetically indistinguishable.

The discrimination assay method here described exploits the distinctive metal ion dependence displayed by individual mNMNATs, and introduces for the first time a matrix substitution calculation to obtain simultaneously the unknown activity values of the three isozymes. After assessing the reliability of the method using reconstituted mixtures of pure recombinant isozymes, the procedure was tailored for assaying NMNAT isozymes in tissue extracts from mouse. As this species is frequently used to model human conditions, the ability to discriminate differential contributions to NAD synthesis in physio-pathological conditions is particularly useful.

Main advantage of this method is that it allows quantitative determination of the activity of the isozymes simultaneously present in a given sample, without their prior physical separation. Isozymes separation, such as one relying on their subcellular compartmentation, *e.g.* by differential centrifugation, would be not suitable for quantitative measurements essentially due to 1) cross-contamination of fractionated particles, 2) not exclusive compartmentation of isozymes, which can be present in more than one subcellular district [43], [44], 3) incomplete recovery of a given compartment due to particle rupture, 4) intrinsic isozyme instability (*e.g.*, NMNAT2 [36]). On the other hand, use of this method can be instrumental to assess the level of different isozymes simultaneously present in subcellular fractions obtained by different fractionation methods, as well as to appraise the recovery of individual isozymes following a fractionation step, and thus for the quantitative comparison of different fractionation methods.

A fundamental requirement of this method is the preliminary selection of appropriate assay conditions for discrimination, encompassing biochemical, analytical, and mathematical features. Biochemical features refer to the selection of assay mixtures leading to consistently dissimilar isozyme reaction rate values. Analytical requirements means that actual rate values should be relatively high, in order to minimize the intrinsic experimental error. The mathematical conditions dictate that the determinant of the matrix employed is non-zero. Our selected conditions depicted in Fig. 3 have been devised taking into account all these features, leading to reliable and reproducible results within good confidence limits, as reported in Tables 2 and 3. It can be noted that, for matrix calculation, the internal normalization with respect to recombinant isozyme rates minimizes experimental variance of measured rate values, caused by factors such as freshness of reagents. Thus, greater accuracy is achieved when activity ratio coefficients for matrix calculation are determined on an individual-experiment basis, or each time reagent stock solutions are renewed. The convenience of this method is improved by the observed stability of the recombinant mouse isozymes (Fig. 2).

Compared to the previously described discrimination assay [13], this method has the advantage that it bypasses the need for only one isozyme to be active in each selected assay condition. In this view, it can be seen as an extension of the previous methodology, whose enhanced statistical accuracy arises from the matrix construction from multiple data points. It can be further improved by selecting discrimination assay conditions positively affecting the biochemical, analytical, or mathematical features of the method. It is also amenable to future custom-adaptation, *e.g.* by replacing HPLC with high-throughput NAD quantitation based on microplate readers and fluorescence cycling assays. Finally of course, it could be extended to mammalian systems other than human and mouse, and to any other enzyme existing in multiple isoforms (even four or more). The only requirement is to establish a number of assay conditions that differentially favour the various isoforms, yielding an appropriate number of simultaneous equations for matrix calculation.

Efforts to elucidate the role played by the three mammalian NMNAT isozymes in NAD biosynthesis have included a number of expression and immunological studies on their tissue distribution and cellular localization [11], [12], [14], [44]. However, as activity levels may differ from both mRNA and protein levels, the assessment of the individual contributions to compartmentalized NAD biosynthesis was hampered by the inability to simultaneously assay individual isozyme activities in crude cell extracts, thus limiting the significance of the above data. This method is intended to fill this gap. Our previous report first allowed a direct determination of individual NMNAT activities in human brain and liver tissue extracts [13]. In this work, the improved discrimination assay yielded similar data using these same tissues from mouse suggesting similar distribution patterns of isozyme activity and validating mouse as a model for compartmentalized NAD-biosynthesis in NAD-related human conditions and diseases in these tissues. The main difference is the activity of NMNAT2, which is comparable to NMNAT1 in mouse whole brain (Fig. 4A and 5A), but predominant over other activities in the human brain [13]. However, the human sample was a brain peritumoral specimen, so it remains to be determined how closely this represents the normal condition. Alternatively, it may reflect the greater contribution of white matter, and hence NMNAT2-rich axons, to human brain. Furthermore, the activity profile in mouse brain does not contradict the relatively higher expression level of NMNAT2 (Fig. 4B), given that its catalytic efficiency is way lower than NMNAT1.

Furthermore, comparison of wild-type and Wld^S^ tissues by the present discrimination assay revealed that the expected increase in total NMNAT activity in Wld^S^ [40] was accounted for by only isoform 1 activity, encompassing the indistinguishable contributions by both mNMNAT1 and Wld^S^ species. The activity of the two other isozymes was unchanged in mutant tissues with respect to wild-type, thus ruling out the occurrence of compensatory changes in mNMNAT2 and mNMNAT3 expression. This is consistent with independent, non-redundant functions of these extra-nuclear NMNAT isozymes. Interestingly, in the opposite case of ∼50% NMNAT1 activity reduction, as observed in whole brain from heterozygous NMNAT1 null mice, this method showed that compensatory changes were similarly absent [2]. Indeed, the activities of mNMNAT1 and Wld^S^ are not discriminated by our assay method, as we found no assay conditions leading to consistently dissimilar reaction rates. Nonetheless, their respective individual contribution could be determined by comparing isoform 1 individual activity in wild-type and Wld^S^ tissues. Alternatively, they could be likewise determined by comparing overall NMNAT activity in wild-type and Wld^S^ tissues, once unchanged mNMNAT2 and mNMNAT3 contributions have been assessed by means of the discrimination assay. In both cases, effective profiling of all three endogenous mNMNATs and Wld^S^ enzyme activities could be achieved in Wld^S^ mutant tissues. As an example, from data in Fig. 5, the Wld^S^ contribution to total NAD synthesis can be estimated to account for ∼0.4 mU/mg in brain, and ∼0.55 mU/mg in liver. Though partly indirect, this evidence is only made possible by the application of the present discrimination assay.

In conclusion, we have developed a novel method of wide applicability to discriminate the individual activity of mammalian NMNAT isozymes. Its preliminary application validated the study of NAD biosynthesis in mouse tissues as a model for human diseases. In particular, the method enables assessment in mouse brain and liver of all known NMNAT reaction-catalysing isozymes, including mNMNAT1, mNMNAT2, mNMNAT3, and Wld^S^. Additional preliminary experiments suggest this assay can also be applied to mouse nerve tissue extracts (unpublished). The ability to analyse individual isozyme fluctuations in a number of physiological and pathological conditions will be important for understanding the mechanism by which localized NMNAT catalysis specifically contributes to axon viability, as well as in other NAD-related conditions such as ageing and metabolic disorders.

## Supporting Information

Table S1 **Synthetic oligonucleotides used in this study.**
(DOCX)

Table S2 **Composition of a typical set of discrimination assay mixtures.**
(DOCX)

Methods S1 **Example of simultaneous single-sample determination of NMNAT isozyme activities in mouse tissues.**
(DOCX)

Methods S2 **MS Excel spreadsheet used for matrix calculation.**
(XLSX)
