# Supplementary material for: Simultaneous Single-Sample Determination of NMNAT Isozyme Activities in Mouse Tissues
Source: PLoS One. 2012 Dec 31;7(12):e53271. doi: 10.1371/journal.pone.0053271 (PMC3534050; doi:10.1371/journal.pone.0053271)
Supplement: Table S1 — Synthetic oligonucleotides used in this study. (DOCX) [file pone.0053271.s001.docx]

**Table S1: Synthetic oligonucleotides used in this study**

| **Cloning of Mouse NMNATs** | | |
| --- | --- | --- |
| *Primer name* | *Primer sequence (5′-3′)* | *Description* |
| mAT1F28c | ATTTGA*CATATG*GACTCATCCAAGAAGA | 5' primer for cloning m*NMNAT1* |
| mAT1R28c | ATT*GAATCC*TCACAGAGTGGAATGGTGG | 3' primer for cloning m*NMNAT1* |
| mAT2F28c | ATTTGA*CATATG*ACCGAGACCACAAAGAC | 5' primer for cloning m*NMNAT2* |
| mAT2R28c | ATT*AAGCTT*CTAGCCCGAGGCGTTGATG | 3' primer for cloning m*NMNAT2* |
| mAT3F28c | ATTTGA*CATATG*AAGAACCGAATCCCTG | 5' primer for cloning m*NMNAT3* |
| mAT3R28c | ATT*AAGCTT*CTAGCCAGTCTTTCCTTTCC | 3' primer for cloning m*NMNAT3* |
| Wld^S^F28b | ATTTGA*GCTAGC*ATGGAGGAGCTGAGCGCTG | 5' primer for cloning *Wld^S^* |
| Wld^S^R28b | ATT*GTCGAC*TCACAGAGTGGAATGGTTG | 3' primer for cloning *Wld^S^* |
| **Real-time PCR Analysis (relative quantification)** | | |
| *Primer name* | *Primer sequence (5′-3′)* | *Description* |
| mAT1F | TTCAAGGCCTGACAACATCGC | 5' primer for *mNMNAT1* |
| mAT1R | GAGCACCTTCACAGTCTCCACC | 3' primer for *mNMNAT1* |
| mAT2F | CAGTGCGAGAGACCTCATCCC | 5' primer for *mNMNAT2* |
| mAT2R | ACACATGATGAGACGGTGCCG | 3' primer for *mNMNAT2* |
| mAT3F | GGTGTGGAGCTGTGTGACAGC | 5' primer for *mNMNAT3* |
| mAT3R | GCCATGGCCACTCGGTGATGG | 3' primer for *mNMNAT3* |
| BACTF | TGTTACCAACTGGGACGACA | 5' primer for *beta-ACTIN (ref. gene)* |
| BACTR | GGGGTGTTGAAGGTCTCAAA | 3' primer for *beta-ACTIN (ref. gene)* |

Primer overhangs are underlined and restriction sites are italicized. Start codons are in green, stop codons in red. A brief description of what each primer was used for is provided.
