# Supplementary material for: Simultaneous Single-Sample Determination of NMNAT Isozyme Activities in Mouse Tissues
Source: PLoS One. 2012 Dec 31;7(12):e53271. doi: 10.1371/journal.pone.0053271 (PMC3534050; doi:10.1371/journal.pone.0053271)
Supplement: Table S2 — Composition of a typical set of discrimination assay mixtures. (DOCX) [file pone.0053271.s002.docx]

**Table S2: Composition of a typical set of discrimination assay mixtures**

| **Assay Reagent** | **Mix “*A*”** | **Mix “*B*”** | **Mix “*C*”** | **Mix “*D*”** | **Final [ ]** |
| --- | --- | --- | --- | --- | --- |
| HEPES/KOH 0.1 M, pH 7.5 | 120 μL | 120 μL | 120 μL | 120 μL | 30 mM |
| BSA 10 mg/mL | 24 μL | 24 μL | 24 μL | 24 μL | 0.6 mg/mL |
| NaF 0.2 M | 40 μL | 40 μL | 40 μL | 40 μL | 20 mM |
| ATP 12.5 mM | 32 μL | 32 μL | 32 μL | 32 μL | 1 mM |
| DTT 50 mM | 8 μL | 8 μL | 8 μL | - | 1 mM |
| MgCl_2_ 1 M | 10 μL | - | - | - | 25 mM |
| MgCl_2_ 1 mM | - | 20 μL | - | - | 50 μM |
| ZnCl_2_ 10 mM | - | - | 60 μL | - | 1.5 mM |
| CoCl_2_ 25 mM | - | - | - | 64 μL | 4 mM |
| Protein sample | brain extract (70 μL)  (*) recombinant mNMNATs (15-47 μL) | | | | 1.06 mg/mL  0.15-2.40 μg/mL |
| H_2_O | up to 392 μL final volume | | | | - |
| NMN 50 mM (reaction start) | 8 μL | 8 μL | 8 μL | 8 μL | 1 mM |

(*) pure preparations of recombinant mNMNATs were thawed from -20 °C, rapidly treated with Chelex (see Methods), and diluted just before their use in assay buffer to final 1.6 μg/mL (mNMNAT1), 34.8 μg/mL (mNMNAT2), and 20.8 μg/mL (mNMNAT3).
