# Supplementary material for: Simultaneous Single-Sample Determination of NMNAT Isozyme Activities in Mouse Tissues
Source: PLoS One. 2012 Dec 31;7(12):e53271. doi: 10.1371/journal.pone.0053271 (PMC3534050; doi:10.1371/journal.pone.0053271)
Supplement: Methods S1 — Example of simultaneous single-sample determination of NMNAT isozyme activities in mouse tissues. (DOCX) [file pone.0053271.s003.docx]

**Methods S1: Example of simultaneous single-sample determination of NMNAT isozyme activities in mouse tissues.**

The stepwise routine described below was carried out on a crude extract from wild-type mouse brain (C57BL/6).

*Step 1: preparation of assay mixtures*

Four kinds of mixtures (400 μL final volume) were prepared according to the scheme in supplemental Table S2, and appropriately replicated to allow parallel assaying of the three recombinant mNMNAT isozymes and the freshly-made brain extract (see below). All mixtures, only missing NMN substrate and brain extract, were kept on ice until addition of these last components.

*Step 2: tissue extract preparation*

Brain tissue (~50 mg) was rapidly homogenized in liquid N_2_, extracted by sonication in 10 vol of appropriate buffer, and treated with Chelex resin as described in Methods. The resulting metal-free brain extract was added (70 μL, 426 μg protein) to the corresponding mixtures above kept on ice.

*Step 3: reaction running and C18-HPLC analysis*

After short pre-warming, all assay mixtures were supplemented with NMN to start the reaction and placed in a water-bath at 37 °C. After 5 min, 10 min, and 15 min incubation, 100-μL aliquots were collected and stopped by rapid mixing with 50 μl ice-cold 1.2 M HClO_4_. After short ice-storing and centrifugation (5 min, 16,000 × *g*), 135-μl supernatant aliquots were neutralized by adding 28 μl of 1 M K_2_CO_3_, centrifuged again to remove insoluble KClO_4_, and directly injected onto C18-HPLC. For each assay, the area of NAD peak product at each incubation time was then integrated and recorded.

*Step 4: data processing and matrix calculation*

The HPLC integrated NAD peak areas recorded were inserted into a pre-formatted MS Excel spreadsheet (red boxed cells in the attached file “Methods S2”) together with the indicated “calculation factors”, thus allowing overall automatic calculation in one-step and yielding the final activity of each isozyme in the brain extract.

The calculation procedure is summarized below; it can be seen in the spreadsheet formulas used. For each assay mixture, the NAD area versus time was first evaluated by linear regression (R^2^ value close to unity) and then the slope (NAD area/min) was used to calculate the corresponding reaction rate. The conversion of areas into nanomoles was made by application of the Lambert-Beer’s law: the area values, expressed as ‘μAU*sec’ due to the software used (Shimadzu LCsolution v 1.24), were first converted into ‘mAU*mL’ by appropriate factoring, and then into nanomoles dividing by the NAD millimolar extinction coefficient at 260 nm (18.6 mM^-1^ cm^-1^). From these values, taking into account dilution factors and protein concentration, the NMNAT specific activities were finally calculated and expressed as nanomoles min^-1^ mg^-1^ (*i.e.* mU/mg). The use of specific activity is preferable for better comparing different samples but is not mandatory for calculation.

In the spreadsheet left panel (“Recombinant mNMNATs assays for matrix construction”), the reaction rates of the three recombinant NMNATs under conditions “B”, “C”, and “D” were divided each by the reference activity “A”, yielding a first matrix construction including the nine reaction rate ratios (matrix coefficients b1, c1, d1; b2, c2, d2; b3, c3, d3). From this, the inverse matrix and the matrix determinant were next built by using the Excel functions MINVERSE and MDETERM, respectively (see Methods). The matrix determinant in this example is 0.304 (absolute value).

In the spreadsheet right panel (“Tissue extract assays and matrix calculation”) the tissue activity values are calculated as above by assaying the brain extract under conditions “B”, “C”, and “D”. Their multiplication to the inverse matrix through the Excel function MMULT (see Methods) yields the final matrix-calculated values of 0.095 mU/mg for mNMNAT1, 0.070 mU/mg for mNMNAT2, and 0.004 mU/mg for mNMNAT3, all referred to the reference assay condition “A”.
